# Leukocyte telomere dynamic change in patients with mild to moderate covid-19 during three weeks of follow-up: Relation with therapy

**DOI:** 10.5937/jomb0-63678

**Published:** 2026-03-17

**Authors:** Jelena Kotur-Stevuljević, Milenković Marina Roksandić, Jelena Vekić, Nemanja Dimić, Dejan Dimić, Danica Ćujić, Azra Guzonjić, Marija Gnjatović, Aleksandra Todorović, Nataša Bogavac-Stanojević

**Affiliations:** 1 University of Belgrade, Faculty of Pharmacy, Department for Medical Biochemistry, Belgrade; 2 Municipal Institute for Lung Diseases and Tuberculosis, Belgrade; 3 University of Belgrade, Faculty of Medicine, University Clinical-Hospital Center Dr Dragisa Misovic-Dedinje, Belgrade; 4 University of Belgrade, Institute for the Application of Nuclear Energy INEP, Belgrade

**Keywords:** COVID-19, oxidative stress, telomere length, biomarker, vitamin D, COVID-19, oksidativni stres, dužina telomere, biomarker, vitamin D

## Abstract

**Background:**

A three-year COVID-19 pandemic revealed aspectrum of disease severity and clinical manifestations. Themost intriguing part of this phenomenon lays in inter-individual variability in COVID-19 course among patients,which is attributable to patient's age, comorbidities and general health status. Focus of this follow-up study was toassess leukocyte telomere length change in mild-to-moderate COVID-19 patients and concomitant influence ofinflammation, oxidative stress (OS), pulmonary involvementand implemented therapy on the course of the disease.

**Methods:**

Routine biochemical/haematological parameters, markers of OS (prooxidants and antioxidants), vitaminD, IgM and IgG antibodies level and relative length ofleukocyte telomeres (rLTL) were measured at three timepoints (at diagnosis, after 14 and 21 days from the diseaseonset) in blood samples of 31 consecutive COVID-19patients, with a mild (n = 16) and moderate (n = 15) form ofthe disease, treated on an outpatient basis.

**Results:**

Although the patients had reduced rLTL at baseline (median: 0.592; 25th - 75th percentiles: 0.518­0.724), it significantly increased during the follow-up(median: 0.773; 25th - 75th percentiles: 0.615-0.923; P&lt;0.01). The rate of telomere attrition was associated withthe extent of OS and pulmonary involvement. During follow-up, the burden of OS was reduced, while antioxidantdefence mechanisms were recovered. The use of antibiotics and N-acetylcysteine-propolis supplementation wasassociated with telomere lengthening.

**Conclusions:**

Results of this study revealed significant interaction between OS, inflammation and leukocyte telomerelength attrition in COVID-19. Our data suggest that rRTLcan be a biomarker that enables more precise therapy decision and accurate patient status estimation.

## Abstract (sr)

**Uvod:**

Tokom trogodišnje pandemije COVID-19 otkrivenoje da postoji čitav spektar težine bolesti i kliničkih manifestacija. Najintrigantniji deo ovog fenomena leži uinterindividualnoj varijabilnosti toka COVID-19 među pacijentima, što se može pripisati starosti pacijenata, komorbiditetima i opštem zdravstvenom stanju. Fokus ove studijepraćenja bio je da se proceni promena dužine telomeraleukocita kod pacijenata sa blagim do umerenim COVID19 i istovremeni uticaj inflamacije, oksidativnog stresa(OS), zahvaćenosti pluća i sprovedene terapije na tokbolesti.

**Metode:**

Rutinski biohemijski/hematološki parametri,markeri OS (prooksidanti i antioksidanti), nivo antitela navitamin D, IgM i IgG i relativna dužina telomera leukocita(rLTL) mereni su u tri vremenske tačke (pri postavljanjudijagnoze, posle 14 i 21 dana od početak bolesti) u uzorcima krvi 31 pacijenta sa COVID-19, sa blagim (n = 16) iumerenim (n = 15) oblikom bolesti, lečenim ambulantno.

**Rezultati:**

Iako su pacijenti imali smanjenu rLTL na početku(medijana: 0,592; 25. - 75. percentil: 0,518-0,724), onase značajno povećavala tokom praćenja (medijana: 0,773;25. - 75. percentil: 0,615-0,923; P&lt;0,923). Stopa skraćivanja telomera bila je povezana sa obimom OS i zahvaćenosti pluća. Tokom praćenja, opterećenje OS je smanjeno,dok su antioksidantni odbrambeni mehanizmi oporavljeni.Upotreba antibiotika i suplementacije N-acetilcisteinpropolisom bila je povezana sa produženjem telomera.

**Zaključak:**

Rezultati ove studije otkrili su značajnu interakciju između OS, upale i smanjenja dužine telomeraleukocita kod COVID-19. Nasi podaci ukazuju da rRTLmože biti biomarker koji omogućava precizniju odluku oterapiji i tačnu procenu statusa pacijenta.

## Introduction

The COVID-19 enigma is not solved yet, either from clinical or pathological points of view. In order to gain a better understanding of the disease, its consequences and therapeutic possibilities, many scientific groups investigate biomarker aspects of the disease. A spectrum of subjects' individual reactions to SARS-COV-2 viral load leading to mild, moderate, severe or even fatal disease is still unresolved. Disease heterogeneity could be a consequence of innate immune system operability diversity in different subjects [1]. The so-called "cytokine storm", a hallmark of immune system excessive activation, is a cause of following oxidative stress storm, which is already has been proposed by Kosanović et al. [2] and also confirmed in our laboratory [3].

It is well known that infectious diseases cause telomere shortening, and initial telomere length could predict resistance to the disease [4]. However, different studies have investigated telomere length in relation to COVID-19 infection, but presumably in severe forms of disease and without following telomere length dynamic change during the disease duration. According to presently available data, telomere in COVID-19 has the potential to be a predictor of disease severity, progression and outcome, so that could be a useful indicator for the treatment direction [5].

Regarding the deep involvement of the immune system in COVID-19-related disease course, frequent over-activation of inflammatory response, followed by OS eruption [6][7], our study intended to estimate OS evolution and patients' clinical status in mild and moderate COVID-19 disease patients. Oxidative stress is considered a key cause of telomere attrition because its special vulnerability towards oxidative stress damage. The special susceptibility of the telomeres lays in its high guanine content. Among the four DNA bases, guanine has the lowest redox potential, making it the most susceptible to oxidation [8]. Armstrong meta-analysis [9] confirmed the deleterious influence of oxidative stress on telomere attrition in vivo, while connecting these two features with ageing.

Primarily we aimed to assess the influence of inflammation and OS, so as mutual involvement of inflammation and thrombosis on leukocyte telomere length as biomarkers of biological age (thus vitality of organism and its capability to combat infection), and also as a marker of the disease. Moreover, we wanted to follow the therapy effect on leukocyte telomere length (LTL) during the 21 days of patients' follow-up until complete recovery.

## Materials and methods

### Patients

This study included 31 consecutive COVID-19-confirmed patients (PCR and/or serology tested) from the Municipal Institute for Lung Diseases and Tuberculosis, Belgrade, Serbia, one of the COVID-19 centres during pandemics. The ethical Committee of the Institution approved the study (Ethical approval No. 366/2, February 2022.). Patients' recruitment lasted during March 2022, and follow-up was scheduled on the 14th and 21st day from the onset of the symptoms for the patients who signed informed consent for entering the study. Diagnostics regarding initial COVID-19 confirmation, biochemical/haemato-logical parameters panel, chest X-ray to clinical staging assessment, and therapy prescription were performed according to the actual protocol of the Ministry of Health of the Republic of Serbia. For the classification of patients, we adopted the criteria established by the National Institute of Health, USA. According to these criteria, patients were categorized into two groups: mild and moderate. Mild illness is characterized by the presence of common COVID-19 symptoms, including fever, cough, sore throat, malaise, headache, muscle pain, nausea, vomiting, diarrhoea, and loss of taste and smell. However, individuals with mild illness do not experience shortness of breath, dyspnoea, or show abnormal chest imaging. On the other hand, moderate illness refers to individuals who exhibit signs of lower respiratory disease based on clinical assessment or imaging. Furthermore, their oxygen saturation levels, as measured by pulse oximetry (SpO2), are equal to or greater than 94% while breathing room air at sea level. By utilizing these criteria, we were able to accurately classify our patients into distinct groups, enabling us to better understand and analyse their clinical conditions [10]. Almost half of the patients (15/31, 48%) had pneumonia, while the second half had no specific findings. The patients categorized as pneumonia had symptoms of respiratory infections and chest X-ray verified pneumonia. In our study, we employed the CURB-65 score as a validated clinical prediction rule to assess mortality risk in cases of community-acquired pneumonia and infections at any site. Endorsed by the British Thoracic Society, the CURB-65 score serves as a valuable tool for evaluating the severity of pneumonia [11]. By utilizing these criteria, clinicians can accurately assess the severity of pneumonia and make well-informed decisions regarding patient management and treatment. In our cohort, all patients scored between 0 and 2 on the CURB-65 scale, indicating their suitability for ambulatory treatment. A higher score would suggest the need for hospitalization. Patients with confirmed pneumonia received antibiotics according to the protocol. Detailed list of all implemented antibiotic drugs are given at the Table 1. Antiviral therapy administered in our group of patients was molnupiravir (it was given in 25 patients) [8]. Regarding supplementation, all patients were advised to use vitamin C (2 g/day) and vitamin D (2000 IU/day), while N-a cetyl cysteine (NAC)-propolis (600 mg/day Abela Pharm, Belgrade, Serbia) was advised according to physicians' judgement of patients' clinical performance.

**Table 1:** Socio-demographic and clinical characteristics of the study population. #Asthma, COPD, depression, anxiety, urinary tract disease, GIT, allergies, bronchiectasis,obesity, acute myocardial infarction, thrombophlebitis, varicosities)<br>*azythromicine, levofloxacin, cefixime, moxifloxacin, amoxicillin clavulonate or clarithromycin

Parameter	Baseline (n=31)
Age, years	52 ± 3
Gender, m/f (n (%))	17/14 (54.8/45.2)
Smoking, no/yes (n (%))	18/13 (58.1/41.9)
Symptoms (no/yes (n (%))	
Headache	12/19 (38.7/61.3)
Fever	3/28 (9.7/90.3)
Cough	9/22 (29.0/71.0)
Expectoration	21/10 (67.7/32.3)
Exhaustion	7/24 (22.6/77.4)
Diarrhoea	27/4 (87.1/12.9)
Anosmia	28/3 (90.3/9.7)
Agues	30/1 (96.8/3.2)
Chest pain	20/11 (64.5/35.5)
Palpitations	21/10 (67.7/32.3)
Tremor	30/1 (96.8/3.2)
Vertigo	26/5 (83.9/16.1)
Hear loss	27/4 (87.1/12.9)
Fatigue	10/21 (32.3/67.7)
Vaccination status, n (%)	
No	9 (29.0)
Yes, 2 vaccine doses	6 (19.4)
Yes, 3 vaccine doses	16 (51.6)
Comorbidities, (no/yes (n (%))	
Hypertension	22/9 (71/29)
Arrhythmias	26/5 (84/16)
Cardiomyopathy	25/6 (81/19)
Diabetes	27 /4 (87/13)
Thyroiditis	24/7 (77/23)
Other#	5/26 (16/84)
Therapy (no/yes (n (%))	
Antiviral (molnupiravir)	6/25 (19/81)
Antibiotic*	5/26 (16/84)
Corticosteroids	21/10 (68/32)
Vitamin C (2 g/day)	0/31 (0/100)
Vitamin D (2000 IU/day)	0/31 (0/100)
NAC-propolis (600 mg/day)	8/23 (26/74)
Aspirin	6/25 (19/81)
Fraxiparin	28/3 (90/10)
NOACs	26/5 (84/16)
Chest X-ray,<br>normal/abnormal, i.e.<br>mild/moderate disease	16/15 (52/43)
sO_2_ (%)	96.9±0.22#

Blood was drawn from the study subjects at entry, and follow-up visits, in the mornings, after the night fasting period. Serum was used for most analyses and EDTA blood sample for rLTL and superoxide anion analysis.

### Markers of oxidative stress

Redox status parameters and leukocyte telomere length were estimated at the Department for Medical Biochemistry (Faculty of Pharmacy, Belgrade, Serbia). The levels of O_2_^-^ were quantified by measuring the rate of reduction of nitroblue tetrazolium, following the method established by Auclair and Voisin [12]. Total oxidative status (TOS) was measured using a spectrophotometric method optimized by Erel, and also in our laboratory [13]. Prooxidant-antioxidant balance (PAB) was determined by a modified PAB test using 0.6% 3,3',5,5'-tetramethylbenzidine (TMB) in DMSO as a chromogen [14]. AOPP was assayed in a 20 mM phosphate buffer at pH 7.4, in reaction with glacial acetic acid and 1.16 M potassium iodide. [15]. Ischemia-modified albumin (IMA) levels were measured in plasma using a modified method based on the work of Bar-Or et al. [16]. MDA concentrations were determined using the thiobarbituric acid-reactive substances (TBARS) assay, as previously described by Girotti MJ et al. [17]. Total antioxidant status (TAS) was measured using a spectrophotometric method employing 10 mmol/L ABTS as a chromogen [18]. Levels of SH-groups were measured using Ellman's method [19]. SOD activity was measured using a slightly modified method by Misra and Fridovich, which depends on the ability of the SOD enzyme to inhibit the autooxidation of epinephrine in an alkaline medium [20]. The enzymatic activity of serum PON1 was measured by kinetic measurements using paraoxon as the substrate, using the Richter and Furlong method [21] slightly modified in our laboratory. For all measurements ILAB 300 plus analyser (Instrumentation Laboratory, Milan, Italy) or the micro-plate reader was used [22].

### Leukocyte's relative telomere length

Genomic DNA for leukocyte telomere length (LTL) determination was extracted from collected samples using a commercial DNA kit (Flexi GENE DNA kit, Qiagen). LTL was determined with modified qPCR and calculated as the relative telomere to single copy gene (T/S) ratio [23]. PCR measurements were carried out on an Applied Biosystems (Waltham, Massachusetts, USA), Real-time pCr 7500.

### Immunochemical tests.

Levels of specific IgG and IgM antibodies against SARS-CoV-2 virus were determined by indirect ELISA test developed and validated by INEP ELISA SARS-CoV-2 IgG and ELISA SARS-CoV-2 IgM tests are based on combination of viral spike and nucleocapsid proteins as antigens, while ELISA SARS-CoV-2 IgG (RBD - S protein) is specifically targeting RBD domain of spike protein. All tests are registered as IVD medical devices at the Medicines and Medical Devices Agency of Serbia (registration numbers 515-02-02373-20-003 and 515-02-02370-21-002).

### Statistical analysis

Variables' distribution normality was tested using Shapiro-Wilks' test, and accordingly, data were presented as means and standard deviations or medians and 25^th^-75^th^ percentile values. Kruskal-Wallis ANOVA and Mann-Whitney U test were used for the inter-group comparison, while Friedman's test and post-hoc Wilcoxon's paired test were used for comparison between three study points. Spearman's nonparametric correlation was implemented to test the correlation between rTL and measured OS parameters. Repeated measures ANOVA (mixed model ANOVA) was used for testing the mutual influence of pulmonary involvement and implemented therapy on rLTL. Summary Oxy score was calculated as a difference between Prooxidant and Antioxidant scores using Z score statistics. Prooxidant score was calculated as mean values of Z scores of two prooxidants, TOS and PAB, while Antioxidant score was mean of TAS and SHG Z scores.

## Results

Sample size was calculated using G-Power software (version 3.1.9.7.) and repeated measures-within factor ANOVA approach. We assumed the effect size was medium (0.25) and the non-sphericity correction was 0.958 (calculated from row data for LTL). Alpha level was set at 0.05 and power of the study at 0.80. Calculated sample size was minimally 29 subjects.

The basic socio-demographic, clinical and therapy-related data are presented in Table 1. COVID-19 patients were middle-aged, more non-smokers than smokers (58 vs. 42%), and the majority (71%) were vaccinated 2 or 3 times. Patients developed many symptoms, including fever, cough, headache, exhaustion and fatigue as the most frequent. All patients had mild to moderate symptoms and remained ambulatory throughout the follow-up period. A certain number of patients (61%) also had one or more comorbidities, among which hypertension, arrhythmias, cardiomyopathy, diabetes and thyroiditis were the most frequent. Almost all patients received some form of treatment, including antiviral, antibiotic and vitamin therapy (as antioxidants and vitamins), and aspirin as non-steroidal anti-inflammatory and anti-aggregatory therapy. Almost half of the patients (15/31, 48%) had pneumonia-related characteristic chest X-ray finding, diagnosed as moderate type of disease, while the second half (16/31, 52%) had no specific finding and diagnosed with mild form of disease.

The average values of all routine laboratory parameters at the study entry were within the reference ranges, except C-reactive protein (CRP) concentrations (data not shown here). These findings were in accordance with the patient's ambulatory status. We didn't repeat these routine analyses because they were not in the pathological range at the beginning of the study.

In general, OS diminishing and antioxidant defence recovery were evident throughout the 3 study points, i.e., from baseline to the second control visit (21^st^ day). The levels of all prooxidants (PAB, TOS) and markers of their activities (AOPP MDA, IMA) significantly fell during follow-up, except superoxide anion which level rose during the study period. At the same time, antioxidants levels (SOD, TAS and PON1) were increasing towards the second control visit suggesting the recovery of antioxidant mechanisms during the study duration. Total SHG concentration showed a variable trend, i.e., a rise at the first control visit (14^th^ day) and a return to statistically lower values at the end of the study.

In order to find a reason for rTL increase during the course of disease we performed Spearman's non-parametric correlation with all measured OS parameters. This analysis revealed significant negative correlation with SHG concentration (at the baseline, p=-0.452, P=0.011), while at the 14^th^ day control our results showed significant positive correlation with SHG (p= 0.389, P=0.037). At the 21^st^ day rTL was positively correlated with SOD activity (p= 0.381, P=0.046). Table 2

**Table 2:** Redox status parameters change during the COVID-19 course.

Parameter	Baseline	14^th^ day	21^st^ day	P	Reference values
TOS (μmol/L)	4.15 (2.23-7.45)	3.50 (2.45-7.65)	1.11 (0.50-2.34)^aaa,bb^	0.001	6-20
O_2_^-^ (μmol/L)	42.5 (26.5-52.5)	46.0 (38.5-56.0)	57.0 (46.0-63.5)^aaa,bbb^	<0.001	12-53
PAB (U/L)	140 (128-167)	117 (99-137)	114 (96-123)^aaa,b^	<0.001	0-80
AOPP (μmol/L)	42.3 (39.4-44.1)	39.2 (35.5-41.0)	35.4 (34.6-37.5)^aaa,bb^	<0.001	9-28
MDA (μmol/L)	2.78 (2.41-3.07)	2.04 (1.33-2.78)	1.52 (1.30-1.81)^aaa,b^	<0.001	0.5-3.0
IMA (ABSU)	0.32 (0.26-0.36)	0.19 (0.15-0.30)	0.14 (0.11-0.16)^aaa,bb^	<0.001	<0.400
TAS (μmol/L)	919 (838-998)	872 (776-948)	947 (866-1036)^b^	0.048	900-1400
SOD (U/L)	106 (83-118)	120 (108-135)	136 (126 -142)^aaa,bbb^	<0.001	90-180
PON1 (U/L)	268 (170-663)	283 (200-651)	308 (218-668)	0.007	200-1080
TAS/TOS ratio	246 (130-412)	258 (112-407)	859 (371-1751)^aa,bb^	0.007	>100
SHG (mmol/L)	0.24 (0.20-0.30)	0.28 (0.24-0.35)	0.26 (0.22-0.30)^b^	0.014	0.350-0.615
Oxy score	0.0 (-0.7-1.0)	-0.4^a^ (-1.8-0.4)	-1.4^aaa,bb^ (-2.0 - -0.8)	0.001	-5.2 - -2.2

We have performed additional comparison of all measured parameters between patients with mild and moderate form of disease and found significantly higher CRP and IMA in moderate compared to mild disease patients, while O_2_^·-^ was significantly lower in moderate disease patients, at baseline. After 14 days of the study, our results showed significantly lower SOD activity and higher MDA and IMA values in moderate compared to mild disease form patients. Analysing OS parameters in the third study point we didn't find any significant difference between mild and moderate subgroups (Table 3).

**Table 3:** Redox status parameters change during the COVID-19 course. Data are presented as median (25^th^-75^th^ percentile); P from Mann-Whitney U test

Parameter	Mild disease	Moderate disease	P
CRP (mg/L) baseline	6.9 (3.0-11.2)	12.0 (6.6-26.5)	0.046
O_2_^-^ (mol/L) baseline	48 (40-54)	35 (21-44)	0.015
IMA (ABSU) baseline	0.283 (0.223-0.336)	0.329 (0.308-0.378)	0.042
IMA (ABSU) 14th day	0.149 (0.143-0.220)	0.303 (0.190-0.321)	0.002
SOD 14th day	133 (120-142)	110 (99-124)	0.005
MDA 14th day	1.52 (1.07-2.00)	2.52 (2.13-2.89)	0.014

The levels of specific anti-SARS-Cov-2 IgM antibodies levels were below cut-off value in the majority of participants, five participants were within grey zone, while only one participant had positive anti-SARS-CoV-2 IgM antibodies during the study course. On the other hand, a significant increase during the three-week study was observed for IgG specific for RBD of SARS-COV-2 Spike protein (Table 4). It is interesting that two patients failed to develop antibodies against SARS-CoV-2.

**Table 4:** Antibody levels change during the COVID-19 course. Data are presented as median (25^th^-75^th^ percentile); ^aa^ P<0.01 vs. baseline; ^b^ P<0.05 vs. 14^th^-day control by Friedman test followed by post-hoc Wilcoxon's paired t-test.

Parameter	Baseline	14^th^ day	21^st^ day	P	Reference values
IgM (index)	28 (28-28)	28 (28-30)	28 (28-31)	0.451	<28 negative<br>29-32 borderline<br>≥33 positive
IgG (index)	74.5 (52.5-84.5)	84.0 (71.5-90.0)	86.5 (67.5-92.5)	0.134	<15 negative<br>16-20 borderline<br>≥21 positive
IgG RBD (index)	53.0 (39.5-81. 0)	86.5 (59.0-91.5)	89.5 (56.0-99.0)^aa,b^	0.017	<15 negative<br>16-20 borderline<br>≥21 positive

**Figure 1:**
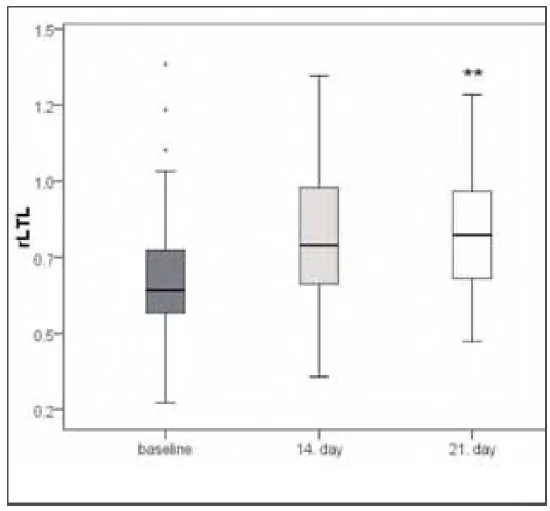
rrLTL at the three study points. **P < 0.01 vs. baseline value according to Friedman's test and Wilcoxon's test as post-hoc test

rLTL was severely decreased in all three study points (median: 0.592 (25^th^ - 75^th^ percentile: 0.518-0.724), median: 0.740 (25^th^ - 75^th^ percentile: 0.612-0.928), median: 0.773 (25^th^ - 75^th^ percentile: 0.615-0.923), respectively), comparing to healthy subjects values from our previous study [11]. The Figure 1 shows that there was an evident rLTL extension during the study (P<0.01 at the third visit vs. baseline).

To test therapy's influence on telomere length recovery, especially in patients with mild and moderate forms of the disease (i.e., with normal and abnormal chest X-ray, respectively), we implemented mixed model ANOVA analysis. This analysis didn't show any difference regarding antivirotic or corticosteroid drugs use (data not shown here). Still, mixed model ANOVA showed longer rLTL in patients who got antibiotics as a therapy. Statistical significance was reached only for patients with normal chest X-ray on 14^th^ day (second visit). The same analysis showed longer rLTL in patients on NAC-propolis supplementation vs. those who didn't use this supplement in both subgroups, with pathological lung changes, so as in a group of patients with normal lung radiography, 21 days from the disease beginning. We noticed a significant fall in rLTL in patients with pathological lung findings who didn't use NAC-propolis from the 14^th^ to the 21^st^ day of the study.

**Figure 2:**
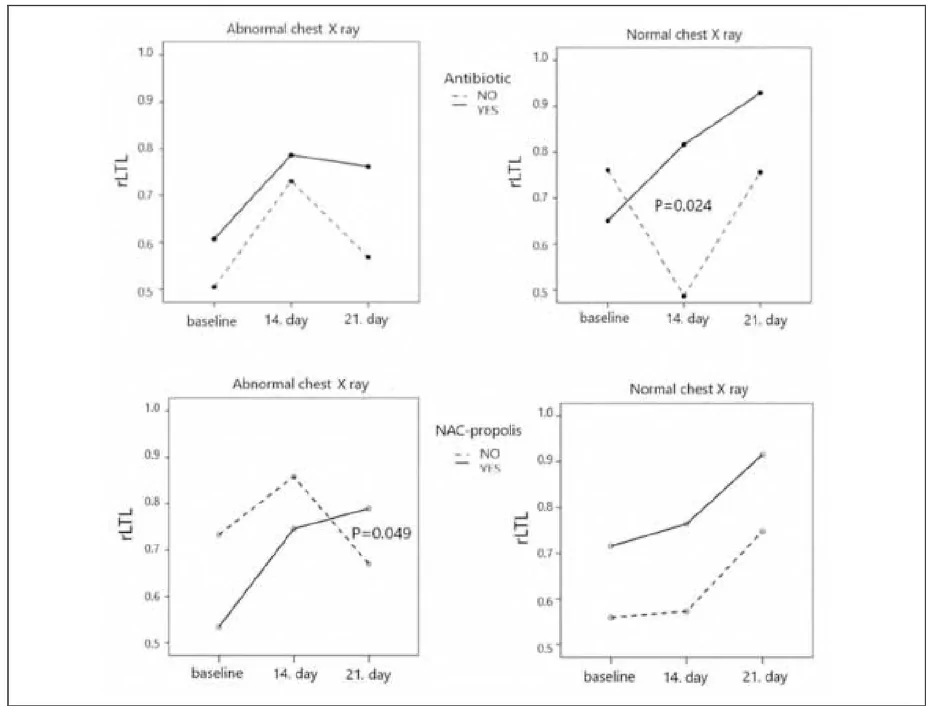
Association of chest X-ray finding and therapy on telomere length recovery during COVID-19 infection.

On the contrary, patients with pathological lung findings who used NAC-propolis experienced continuous rLTL rise. Patients with normal lung findings had a constant increase in rLTL during the study period, regardless of NAC-propolis supplementation. Still, this rise was statistically significant only in the subgroup taking the supplement (Figure 2).

## Discussion

In the presented study, a group of ambulatory COVID-19 patients was followed for 3 weeks starting from the onset of the symptoms. The main idea was to monitor patients' status through the biomarkers' change of both routine and emerging biomarkers (redox status parameters and leukocyte's telomere length). Patients' symptoms and signs of disease, so as implemented therapy protocol was similar to Navarro-Reynoso et al. [24] study and Guntur et al. [25] study.

The antibodies change was in accordance with its dynamic after the infection beginning, similar as in Li et al. [26] study. The levels of IgM were under cut-off value for positive test and remained unchanged during the three weeks of the study duration. Most participants (22/31) were vaccinated with at least two doses, so a significant IgM rise was not expected. On the other hand, all except three participants had sufficient IgG levels against both Spike and Nucleocapsid antigens or solely to RBD, probably due to previous contact with SARS-CoV-2 virus or immunization.

Newly emerged COVID-19 infection stimulated immune cells and provoked IgG rise, particularly IgG specific toward RBD. On the contrary total IgG so as IgG RBD had different dynamic, in our so as in Li's study, the increase of IgG started from beginning and lasted the whole study period in our study, and until 5th week in above-mentioned study. Several groups concluded that unexpected higher IgG compared to IgM concentration at the disease diagnosis point was a consequence of prolonged asymptomatic period of the disease when IgM was first activated, followed with higher IgG activation in the subsequent phases of disease with clear symptoms development [27]. The measurement of anti-SARS-CoV-2 antibodies either in COVID-19 convalescents and/or vaccinated individuals serves as an indicator of humoral immune response following SARS-CoV-2 infection or vaccination. As antibody levels, particularly IgG antibodies against RBD of Spike protein indirectly reflect protective capacity against virus, low antibody titers might be associated with higher risk of infection or worse disease course. The fall in antibody titers is seen in some persons few months after first or later vaccine doses, and decreased specific humoral immunity might be related to higher risk of subsequent infection. Therefore, measurement of anti-SARS-CoV-2 antibodies, either in convalescent or vaccinated individuals may be useful in assessment of immune status and protection against SARS-CoV-2 infection [28][29].

While majority of the routine biochemical and haematological parameters were in reference range, initial concentration of prooxidants and protein and lipid products of its activity in this study was increased, so as their gradual fall upon recovery, which is in accordance with the findings of earlier studies by Lalosevic [3] and Kosanovic [2]. But intriguing were the low baseline concentration of TOS and relatively low superoxide anion level comparable with reference values. Low TOS baseline values, even below the lower reference limit were probably caused by a rapid and sustained reducing blood components increase (non-protein nitrogen compounds), which was already documented for critically ill patients with sepsis and also COVID-19 caused by liver and/or kidney damage or functional disturbance at the peak of infection [30]. Generalized inflammation superimposed to tissue and cells hypo-oxygenation in this kind of fulminant respiratory disease influences mitochondrial metabolism, thus diminishing superoxide anion effusion in the cytosol and out of the cell and blocking its appearance in circulation. This phenomenon is explained by antioxidants, presumably GSH, concentrating during hypoxia [31]. Lower superoxide anion in earlier disease phases could additionally explain low TOS concentration, having in mind that this parameter represents a sum of lipid-hydroperoxides and H_2_O_2_, the former known as the early product of superoxide anion's driven lipid peroxidation and the latter known as a main product of SOD-mediated neutralization of superoxide anion. Significant superoxide anion increases during the three weeks of the study period is probably a consequence of oxygenation improvement due to patients' recovery. In the same time, antioxidative mechanisms were also improved, indicating that redox balance was restored upon recovery. However, it should be noted that deficient oxygenation of COVID-19 patients and superoxide anion increase in the later phase of the disease was suggested to mediate post-COVID reperfusion injury development after more complete blood aeration achievement. Ischemia-reperfusion injury in severe COVID-19 patients as a cause of multiple organ damage was postulated in Ashraf et al. study [32].

Since OS and pro-inflammatory cytokines' coactivation may induce tissues damage in COVID-19, the use of antioxidants is suggested as an adjuvant treatment for faster recovery, which is in accordance with our health care protocols [33]. In line with the previous, the patients included in our study were advised to take antioxidant (vitamin C, NAC, NAC-propolis) and immunomodulatory supplements (vitamin D, zinc), which is already well-known to influence redox status [34].

An intriguing finding of our study is peripheral blood leukocyte telomere attrition, even in patients with milder forms of COVID-19. The rLTL in our COVID-19 patients was significantly shorter when compared to healthy population from our previous study [23]. Although significant increase during the 21 days of study duration was obvious and in line with the improvement of patients' condition, they still had lower rLTL than healthy subjects. It is clear and unequivocally confirmed that shorter telomeres are not just a biomarker of COVID-19 but also could be a good predictor of disease severity because of the opposite correlation of telomere length and disease stadium [35]. Retuerto and associates [36] found relation between shorter LTL and slower radiographic lung abnormalities improvement in a group of severely ill COVID-19 patients, as a consequence of diminished potency of short telomeres to enable tissue recovery, which has already been demonstrated in an animal model lung fibrosis [37]. By testing therapy influence on rate of rLTL recovery in subgroups by different chest X-ray finding a positive impact of antibiotics was revealed. Jin et al. study confirmed positive influence of the azithromycin therapy (the most frequently used antibiotic in this infection) on shorter telomeres recovery in chronic obstructive pulmonary disease patients [38]. This could be explained by confirmed anti-inflammatory and immune-modulatory macrolide drug activity [39] but also with faster general recovery of the patients upon antibiotics influence. Another important finding of our study was that NAC-propolis combination significantly increased rLTL in a group of patients with pathological chest X-ray findings who used this supplement in combination with conventional therapy (Figure 2, Table 4). Positive NAC influence on lung fibrosis is already reported in one meta-analysis, i.e., its efficacy and safety [40] so as of propolis on respiratory tract diseases [41]. NAC's beneficial activity in pulmonary diseases is explained by its direct antioxidant reactivity, its contribution to cysteine and GSH concentration increase, and its mucolytic capability [40]. Propolis' effectiveness in pulmonary diseases is connected with its virucidal/bactericidal activity, inhibition of pathogen entry and more importantly by the immune response modulation (upregulation of pathogen removal and diminishing the extent of inflammation) [41]. There are similar conclusions about NAC and propolis usage during COVID-19 infections regarding its additional positive effects on patients' status improvement and faster recovery [42]. Positive influence of propolis and other bee products on telomere length is already reported [43]. Our study also extends preliminary findings of Soto et al. [44] that NAC influences not only antioxidant system but also positively on LTL.

There are some inherent limitations to our study, including its cross-sectional design and a relatively small study group, suggesting that additional studies with a greater sample size, including severe COVID-19 patients are required. Other factors that might affect redox status, including obesity, dyslipidemia, disturbed glucose homeostasis or kidney function were not considered, since biomarkers of these processes were not routinely assessed in COVID-19 patients, according to the national protocol.

## Conclusion

In conclusion, the results of our study revealed mutual involvement of OS, inflammation and rLTL attrition in COVID-19 pathogenesis and their significant improvement with recovery, at least in mild and moderate COVID-19 patients. By monitoring the dynamics of rLTL change along with the main clinical finding regarding pulmonary involvement and prescribed therapy and supplementation, we were able to reveal delicate changes in telomere length throughout mild and moderate forms COVID-19. The data presented herein supports leukocyte telomere length determination in milder forms of COVID-19 for more precise therapy decision and accurate patients' status estimation. Further research is needed to show whether these findings can be translated to severe COVID-19 patients. During follow-up, all our patients have been recovered, so this study suggests a significant role of antibiotics and antioxidants as important part of management of these patients.

## Dodatak

### List of abbreviations

ALT, Alanine aminotransferase;<br>AOPP Advanced oxidation protein products;<br>AST, Aspartate aminotransferase;<br>CRP, C-reactive protein;<br>IgG, Immunoglobulin G;<br>IgM, Immunoglobulin M;<br>IMA, Ischaemia-modified albumin;<br>LDH, Lactate dehydrogenase;<br>MDA, Malondialdehyde;<br>NAC-propolis, N-acetyl-cysteine-propolis;<br>Propomucil (AbelaPharm, Belgrade, Serbia);<br>O_2_^·-^, Superoxide anion;<br>OS, Oxidative stress;<br>PAB, Prooxidant-antioxidant balance;<br>PON1, Paraoxonase 1;<br>RBD, Receptor binding domain;<br>rLTL, Relative length of leukocyte telomeres;<br>SHG, Total sulfhydryl groups;<br>SOD, Superoxide dismutase;<br>TAS, Total antioxidative status;<br>TOS, Total oxidative status.

### Data availability

The database used and analyzed during the current study are available from the corresponding author upon reasonable request.

### Ethical approval

This study has been approved by the Ethics Committee of the Municipal Institute for Lung Diseases and Tuberculosis, Belgrade, Serbia approved the study (Ethical approval No. 366/2, February 2022.).

### Funding statement

This investigation was supported by the Ministry of Education, Science and Technological Development, Republic of Serbia (Grant Agreement with University of Belgrade-Faculty of Pharmacy No: 451-03-68/2022-14/200161).

### Authors' contributions

Conceptualization, Jelena Kotur-Stevuljević and Marina Roksandić Milenković; Data curation, Jelena Kotur-Stevuljević, Marina Roksandić Milenković, Nemanja Dimić, Dejan Dimić, Azra Guzonjić; Formal analysis, Jelena Kotur-Stevuljević, Marina Roksandić Milenković, Nemanja Dimić, Dejan Dimić, Danica Ćujić, Azra Guzonjić and Aleksandra Todorović; Methodology, Marija Gnjatović, Aleksandra Todorović and Nataša Bogavac-Stanojević; Project administration; Resources, Marija Gnjatović; Writing - original draft, Jelena Kotur-Stevuljević and Danica Ćujić; Writing - review & editing, Jelena Vekić and Nataša Bogavac-Stanojević.

### Conflict of interest statement

All the authors declare that they have no conflict of interest in this work.
